# Vivid imagery is reported faster than weak imagery

**DOI:** 10.1093/nc/niaf054

**Published:** 2026-01-19

**Authors:** Benjy Barnett, Matan Mazor, Giulia Cabbai, Nadine Dijkstra

**Affiliations:** School of Psychological Sciences, Birkbeck, University of London, WC1E 7HX, United Kingdom; Department of Imaging Neuroscience, University College London, WC1N 3AR, United Kingdom; All Souls College and Department of Experimental Psychology, University of Oxford, United Kingdom; Department of Imaging Neuroscience, University College London, WC1N 3AR, United Kingdom; Department of Imaging Neuroscience, University College London, WC1N 3AR, United Kingdom

**Keywords:** vividness, absence, imagery, perception

## Abstract

Visual imagery and external perception rely on similar representations. However, whether the same processes underpin the subjective appraisal of both percepts and mental images is not yet known. One well-known effect in perceptual detection tasks is that people take longer to report perceptions of absence compared to presence. Vividness reports are detection-like in that participants report the presence or absence of a mental image. We therefore asked whether reports of low vividness share commonalities with reports of target absence. Across five pre-existing datasets, we report a robust inverse correlation between imagery vividness ratings and reaction times: participants take longer to report the vividness of mental images when they are weak. In addition, in one of the two datasets that included detection tasks and trait imagery questionnaires we find that individual differences in detection asymmetries (slower responses for absence versus presence in detection tasks) and trait imagery can predict the strength of this vividness-response time relationship. Our results may be suggestive of a shared mechanism employed across both perception and imagery that evaluates the strength of visual experience. Future research is necessary to fully characterize the mechanisms driving this effect.

## Introduction

Early research on the format of mental images used reaction time to show similarities with perception. For example, in their seminal study, Shepard and Metzler showed that participants take longer to compare two differently rotated three-dimensional objects when their rotational angle is larger, similar to rotating those objects in reality (Shepard and Metzler 1971). Relatedly, Kosslyn and colleagues showed that it takes more time to traverse larger distances in a mental image, such as between the feet and the head of an imagined person, versus the feet and the knees, similar to scanning in perception (Kosslyn and Ball 1978). These observations suggested that imagery, like perception, relies on depictive representations, an idea that has now been confirmed with neuroimaging (Siclari *et al*. 2017, Dijkstra *et al*. 2019a, Pearson 2019). However, whilst the representational overlap between imagery and perception is well known, whether appraisal of mental images shares similar cognitive mechanisms to the appraisal of external perception is not yet established. Importantly, we use the term ‘subjective appraisal’ to refer to the evaluation of the subjective strength of percepts or mental images.

One effect consistently exhibited in perceptual detection tasks is an asymmetry in reaction times between ‘target present’ and ‘target absent’ decisions (Meuwese *et al*. 2014; Mazor *et al*. 2020, 2021, 2025; Kellij *et al*. 2021). In other words, participants typically take longer to report when they did not see something compared to when they did. Importantly, this effect is also present when participants rate the visibility of visual stimuli on a graded scale, with absence of visibility taking longer than clearer experiences (Andersen *et al*. 2016). Effects such as these can offer important insights into the cognitive and computational processes underlying perception. For instance, one interpretation of the presence-absence asymmetry is that it reflects an asymmetric evidence accumulation procedure for decisions about presence and absence (Mazor *et al*. 2025).

Here, we describe a similar effect in the domain of visual mental imagery. As with the detection of present and absent stimuli in perceptual tasks, people can report the vividness of their mental imagery from weak or absent imagery to highly vivid imagery. Across five independent datasets, we reveal that reported imagery vividness is negatively correlated with the time it takes participants to report the vividness of their imagery. Like perception, reports of low vividness take longer than reports of high vividness. We further explore whether this effect is related to individual differences in presence-absence asymmetries in perceptual detection decisions and in imagery vividness. We contend that the inverse correlation between vividness and reaction time may be suggestive of a shared mechanism employed across both perception and imagery that evaluates the strength or specificity of visual experience, regardless of whether it is imagined or perceived (Kind 2017; Macpherson 2018; Morales 2023; Fazekas 2024; Sulfaro *et al*. 2024). More generally, we hope that reporting this effect will aid in constraining theories and cognitive models of mental imagery and its relationship to perception.

## Methods and materials

### Datasets

We re-analyzed data from four published datasets (Dijkstra *et al*., 2019b; Cabbai *et al*. 2023, 2024; Dijkstra *et al*. 2025) and one unpublished pilot dataset. In each experiment, participants rated trial by trial vividness of their mental imagery. In Dijkstra *et al*. (2019b), participants performed a binocular rivalry task during which they imagined gratings and rated their imagery vividness prior to a rivalry display. Cabbai *et al*. (2023) consisted of participants imagining fruits or vegetables during an attentional capture task. At the end of each trial participants reported the vividness of their imagery. In Cabbai *et al*. (2024), participants were asked to listen to different sounds and rate the vividness of visual mental imagery that accompanied their listening. The task underlying the unpublished pilot dataset follows closely that of Dijkstra *et al*. 2025. In both these tasks, participants performed both detection and imagery tasks simultaneously. As participants viewed dynamic noise stimuli, they were asked to imagine a particular grating in the noise. At the end of each trial, they were asked to report the vividness of their imagery, and whether a grating had actually been presented or not. There were two differences between the two tasks. Firstly, in Dijkstra *et al*. (2025), the detection target’s onset was gradual, whilst in the unpublished dataset target onset was immediate. The purpose of the pilot study was to observe the effect this change had on participants’ behaviour as our previous work on perception and imagery confusion had always used a gradual presentation scheme (Dijkstra *et al*. 2025; Dijkstra and Fleming 2023)*.* Secondly, the unpublished dataset used a sliding scale from 0 to 100 to report imagery vividness, whilst Dijkstra *et al*. (2025) used a 1 to 4 scale. These differences, alongside the fact Dijkstra *et al*. (2025) took place in an fMRI scanner and the pilot data were collected online (Table 1), likely contributed to the difference in reaction times seen across the two experiments. Table 1 describes each experiment, including the imagined stimuli, the sample size, and the vividness rating scale. Further details of each task can be found within the original publications.

**Table 1:** Experimental details of different datasets.

Experiment	Sample Size	Trials Per Subject	Imagined Stimuli	Vividness Scale	Study Type
Dijkstra *et al.* (2019)	69	220	Red/Blue Gratings	Sliding bar [−150–150]	In Person
Cabbai *et al*. (2023)	121	240	Fruits and Vegetables	[1–5]	Online
Cabbai *et al*. (2024) *Supplementary Pilot Experiment 2*	75	24	Living and Non-Living Phenomena (e.g. seagulls, traffic, dog, airplane, etc.)	[1–5]	Online
Dijkstra *et al.* (2025)	24	384	Left/Right Gratings	[1–4]	In Person (fMRI)
Imagery and Perception Pilot Data (2025)	34	Mean = 252.91; Min = 168; Max = 288	Left/Right Gratings	Sliding bar [0–100]	Online

For each experiment, participants were instructed to report their vividness. However, how this was framed and instructed varied slightly across studies. In Dijkstra *et al*. (2019; 2025) and the Pilot Data, participants completed the (Vividness of Visual Imagery Questionnaire (VVIQ); Marks 1973) before participating in the experiment, which defines vividness as to what extent imagery is ‘as lively and vivid as real seeing.’ We speculate that when participants later rated their imagery in the experiments, they used these same criteria. When participants were prompted to rate their imagery in Dijkstra *et al*. (2019; 2025), participants were explicitly asked ‘how vivid is your imagery, from not vivid to very vivid?’ In the pilot data, this wording was changed slightly to ask ‘how vividly did you imagine the stimulus?’ In Cabbai *et al*. (2023, 2024), participants were instructed to voluntarily conjure vivid visual imagery of a target object as soon as they read its name. They were then prompted to rate their imagery with the question ‘How vivid was the imagery that you experienced when visualizing the object?’ They could respond on a Likert scale from 1 (‘no image at all’) to 5 (‘perfectly clear and vivid as if I was actually seeing it’). It is important to note that the vividness scales participants used to report their vividness were different across studies, yet because we do not perform analyses across studies this should not confound interpretation of our analyses.

### Analyses

Data were analyzed in RStudio 1.4.1106 using R version 4.1.0 and *afex* 1.4–1 (Singmann *et al*. 2012). Following previous literature and recommendations from simulation studies, excessively quick (<200 ms) and slow (>10s) trials were removed, as were trials where reaction times fell 3 SD outside the mean (Berger and Kiefer 2021; Mazor *et al*. 2021; Kimchi *et al*. 2023). For Dijkstra *et al*. (2019) and the Imagery and Perception Pilot Data, the sliding bar scales were split into quartiles, transforming vividness ratings to a 1 to 4 scale within each participant. Linear mixed effects models were constructed for each dataset with the reaction time for imagery vividness ratings as the dependent variable. Individual participants were modelled with random intercepts and slopes for the main effect of vividness on reaction time. In all models, covariates were z-scored prior to estimating the model.

All of our analyses were of an exploratory nature, seeking to assess whether reaction time and vividness ratings covaried reliably across different studies*.* As such, two-sided tests were used across all analyses. To address this question, the first analysis of interest was to test whether there were significant correlations between vividness ratings and reaction times:


$$ \mathrm{RT}\sim \mathrm{vividness}+\left(1+\mathrm{vividness}\ |\ \mathrm{subject}\right) $$

Subsequently, to explore whether this correlation was related either to the asymmetry participants exhibited in detection reaction times or their VVIQ scores, we added these data as covariates into the models above and tested for their interaction with imagery vividness:


$$ \mathrm{RT}\sim \mathrm{vividness}\,\times\, \mathrm{detection}\_\mathrm{asymmetry}\kern0.5em +\left(1+\mathrm{vividness}\ |\ \mathrm{subjID}\right) $$

$$ \mathrm{RT}\sim \mathrm{vividness}\times \mathrm{VVIQ}+\left(1+\mathrm{vividness}\ |\ \mathrm{subjID}\right) $$

The random effects structure in the models above was determined by initially opting for the maximum random effect structure and iteratively reducing it until the models successfully converged (Barr *et al*. 2013; Bates *et al*. 2015). Significant interactions here would suggest that individuals with different asymmetries in detection reaction times, for example, also exhibit varied strength in the relationship between imagery vividness and reaction time. We tested the directionality of this interaction (do participants with greater detection asymmetry show more positive or negative correlations between imagery vividness and reaction time?) with follow up tests of simple slopes, by computing the vividness-RT correlation at different values of detection asymmetry (−1 SD, 0SD, and + 1 SD around the mean). We then visualized these results by binning participants into groups of weak, medium, and strong asymmetries (Fig. 2).

## Results

### Imagery vividness is negatively correlated with reaction time

**Figure 1:**
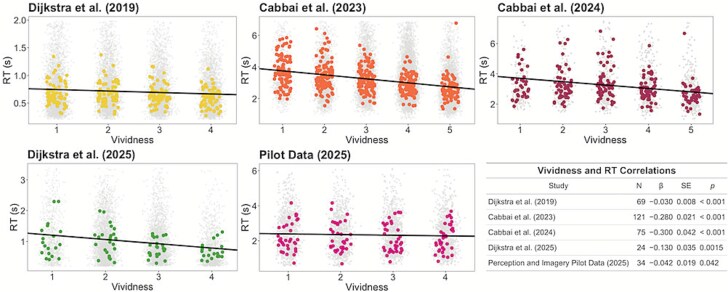
Imagery reaction time is negatively correlated with imagery vividness. Across all five datasets, we observed a significant main effect of imagery vividness on the time taken by participants to report the vividness of their imagery. Random slopes and intercepts were used to model different participants. Coloured dots show median reaction time per vividness rating for each subject. Transparent grey dots represent individual trials. Black lines illustrate multilevel model fits. Right: Summary of mixed model results for each dataset.

Across all five datasets, the vividness of participants’ imagery was negatively correlated with the time taken to report the vividness of imagery (Fig. 1). In other words, it took individuals longer to report imagery vividness when their imagery was less vivid. Statistical results are summarized in Fig. 1 (bottom-right). In all datasets, we observed a significant negative correlation between the vividness of imagery and the time taken to report it (Dijkstra *et al*. (2019): $\beta$ = −0.03, SE = 0.008, *P* < .001; Cabbai *et al*. (2023): $\beta$ = −0.28, SE = 0.021, *P* < .001; Cabbai *et al*. (2024): $\beta$ = −0.30, SE = 0.042, *P* < .001; Dijkstra *et al*. (2025): $\beta$ = −0.13, SE = 0.035, *P* = .0015; Perception and Imagery Pilot Data (2025): $\beta$ = −0.042, SE = 0.019, *P* = .042).

We performed a control analysis to ensure the relationship between imagery vividness and reaction time was not driven by an unequal number of trials across vividness ratings. To do this, within each dataset, we pseudorandomly under sampled trials until the trial count was equal across vividness ratings. The negative correlation remained significant in all datasets (Dijkstra *et al*. (2019): $\beta$ = −0.03, SE = 0.008, *P* < .001; Cabbai *et al*. (2023): $\beta$ = −0.28, SE = 0.019, *P* < .001; Cabbai *et al*. (2024): $\beta$ = −0.21, SE = 0.052, *P* < .001; Dijkstra *et al*. (2025): $\beta$ = −0.13, SE = 0.034, *P* = .0013; Perception and Imagery Pilot Data (2025): $\beta$ = −0.043, SE = 0.020, *P* = .039.

### Relating imagery vividness, reaction time, and detection asymmetries

**Figure 2:**
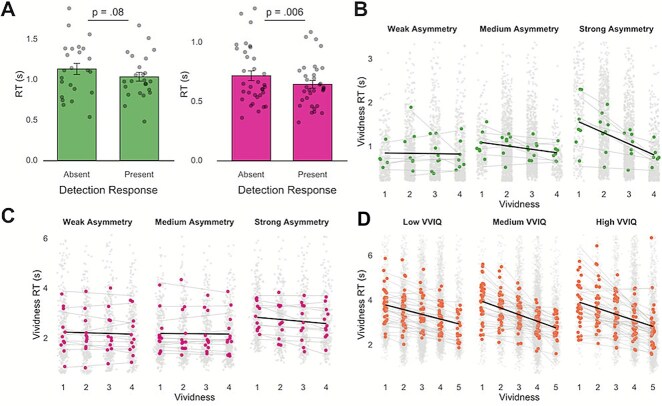
Asymmetries in detection responses and VVIQ interact with imagery vividness and reaction time relationship. (A) Participants generally take longer to report a stimulus was absent than when it was present in two tasks with perceptual detection components. Left: Dijkstra *et al.* (2025); right: Imagery and Perception Pilot Data (2025). (B) Participants in Dijkstra *et al.* (2025) with greater asymmetry between target-present and target-absent detection responses show an increased inverse correlation between imagery vividness and reaction time. Data are binned into weak, medium, and strong detection asymmetry groups for illustration purposes only. (C) The interaction between detection response asymmetry and imagery vividness did not replicate in the Imagery and Perception Pilot Data (2025). (D) Individuals with more vivid trait imagery showed an increased inverse correlation between imagery vividness and reaction time in Cabbai et al. (2023). Data are binned into weak, medium, and strong VVIQ groups for illustration purposes only. Coloured points represent individual subject means. Transparent grey points illustrate individual trials. Transparent grey lines show linear models fit to individual subjects, for visualization purposes only. Black lines reflect linear regression models fit to subject means per each detection asymmetry and VVIQ group, for visualization purposes only.

If the inverse correlation between imagery vividness and reaction time is driven by the same mechanism governing asymmetries in perceptual detection responses, we might expect to see a correlation between these effects over participants, such that participants who show a greater detection asymmetry also show a stronger vividness-reaction time correlation. In two datasets (Dijkstra *et al*. 2025, Perception and Imagery Pilot Data 2025), participants were simultaneously asked to imagine and detect grating stimuli throughout each trial. Before participants reported the vividness of their imagery, they were asked to report whether they saw a grating presented. In line with previous research identifying an asymmetry in reaction times for target-present and target-absent detection responses (Meuwese *et al*. 2014; Mazor *et al*. 2020, 2021, 2025; Kellij *et al*. 2021), there was a general trend for participants to take longer to report that a grating was absent compared to when participants believed it to be present (Fig. 2A, left; Dijkstra *et al*. (2025): Mean_Absent_ = 714 ms, Mean_Present_ = 642 ms, t(23) = 1.82, *P* = .08; Fig. 2A, right; Imagery and Perception Pilot Data (2025): Mean_Absent_ = 1 130 ms, Mean_Present_ = 1 003 ms, t(33) = 2.97, *P* = .006).

To test whether an individual’s asymmetry in detection responses was associated with their asymmetry in vividness judgements, we computed the magnitude of individual participants’ detection asymmetry by subtracting the mean reaction time for target-present responses from the mean reaction time for target-absent responses per participants. In Dijkstra *et al*. (2025), adding individual participant’s detection asymmetry as a covariate in our model of imagery vividness on imagery reaction time again resulted in a main effect of vividness on reaction time ($\beta$ = −0.12, SE = 0.031, *P* < .001) as well as a main effect of detection asymmetry on reaction time ($\beta$ = 0.18, SE = 0.063, *P* = .007), such that people who were slower in target-absent responses in the detection task were overall slower in giving vividness ratings. Importantly, the interaction between detection asymmetry and imagery vividness was also significant ($\beta$ = −0.08, SE = 0.031, *P* = .019; Fig. 2B). To probe this interaction further, we computed simple slopes for the effect of vividness on reaction time at three levels of detection asymmetry: one standard deviation below the mean (−1 SD), the mean (0 SD), and one standard deviation above the mean (+1 SD). This revealed an increasing strength of the vividness-reaction time relationship as participants’ detection asymmetries grew (−1 SD: $\beta$ = −0.04, SE = 0.044, *P* = .33; mean: $\beta$ = −0.12, SE = 0.031, *P* = .0001; +1 SD: $\beta$ = −0.20, SE = 0.043, *P* < .0001). In other words, participants who took longer to report target absence (compared to presence) in perception also took longer to report weak (compared to vivid) imagery. The detection asymmetries entered into these analyses were fully continuous. However, for visualization purposes only, we binned participants’ asymmetry into three quantiles: weak, medium, and strong and plotted the relationship between vividness and reaction time for each (Fig. 2B). Binning was performed using the R tidyverse ntile() function, which split participants into three equally sized groups based on their detection asymmetries. Binned data were for visualization purposes only and were never entered into analyses. Adding participants’ detection asymmetries as a covariate to our model for the Imagery and Perception Pilot Data (2025) also revealed a significant main effect of vividness on imagery reaction time ($\beta$ = −0.04, SE = 0.02, *P* = .039), however the main effect of detection asymmetry ($\beta$ = 0.25, SE = 0.131, *P* = .066) and the vividness x asymmetry interaction ($\beta$ = −0.03, SE = 0.02, *P* = .134) were not significant (Fig. 2C). In both datasets, using participants’ median reaction times to compute their detection asymmetry did not influence the statistical significance of the vividness × detection asymmetry interaction term (Dijkstra *et al*., 2025: $\beta$ = −0.08, SE = 0.03, *P* = .012; Pilot Data: $\beta$ = −0.03, SE = 0.021, *P* = .175), establishing that outliers in participants’ detection reaction times were not driving or masking this result.

To ascertain whether participants’ arousal levels were driving response time asymmetries, we compared reaction times in trials where stimuli were presented compared to those where they were absent (as opposed to trials where participants reported presence or absence). If the asymmetry between ‘absence’ and ‘presence’ responses is driven entirely by arousal, this asymmetry should be abolished when contrasting when a stimulus was actually presented or not. This is because, in the latter case, the trial order is orthogonal to participants’ arousal levels. In both datasets that contained a detection component (Dijkstra *et al*. 2025; Pilot Data 2025), we found that reaction times were significantly slower on absent versus present trials (Dijkstra *et al*. 2025: mean difference = 49 ms, 95% CI [6 90], t(23) = 2.35, *P* = .02; Pilot Data (2025): Mean difference = 68 ms, 95% CI [22113], t(33) = 3.05, *P* = .004), suggesting that arousal cannot entirely explain reaction time asymmetries in detection.

### Relating imagery vividness, reaction time, and trait imagery

A further interesting question is whether an individual’s trait imagery impacts the relationship between vividness judgements and reaction times. For example, it might be the case that, compared to individuals with weak imagery, people with very vivid imagery expect their mental images to be vivid. This could facilitate reports of high-vividness since such experiences would align with their prior expectations. This might then lead such individuals to report vivid imagery faster than weak imagery compared to individuals with lower trait imagery. To explore whether participants’ self-reported trait imagery strength [as measured by VVIQ (Marks 1973)] was related to the relationship between imagery vividness and reaction time, we added participants’ VVIQ scores to the model for two datasets (Cabbai *et al*. 2023, 2024). In Cabbai *et al*. (2023), adding VVIQ as a covariate resulted in a significant main effect for vividness on reaction time ($\beta$ = −0.27, SE = 0.22, *P* < .001) and a significant interaction between vividness and VVIQ ($\beta$ = −0.06, SE = 0.023, *P* = .012, Fig. 2D). Computing simple slopes at different levels of VVIQ showed the vividness and reaction time relationship to increase in strength as VVIQ increased (−1 SD: $\beta$ = −0.22, SE = 0.034, *P* < .0001; mean: $\beta$ = −0.28, SE = 0.022, *P* < .0001; +1 SD: $\beta$ = −0.34, SE = 0.030, *P* < .0001). These results demonstrate that as participants’ trait imagery increases, the relationship between imagery and reaction time becomes more negative. The main effect of VVIQ on imagery reaction time was not significant ($\beta$ = 0.006, SE = 0.068, *P* = .92), meaning trait imagery did not correlate with the general speed of participants’ responses. For illustration purposes, we binned participants into three quantiles for low, medium, and high VVIQ scores and plotted the relationship between imagery vividness and reaction time for each quantile (Fig. 2D). To confirm that this effect was not a result of people with high VVIQ scores reporting weak imagery on fewer trials, we binned trial-based vividness ratings into low, medium, and high vividness within each subject and balanced trial numbers within each of these bins by pseudorandomly undersampling trials in each bin to match the bin with the lowest trial count. This effectively removes the impact of different trial counts for different vividness ratings between people with low and high VVIQs. Running the model on this balanced data again revealed a significant interaction ($\beta$ = −0.03, SE = 0.011, *P* = .015), demonstrating that unequal trial numbers were not driving the interaction between VVIQ and vividness. Adding VVIQ as a covariate to the model for Cabbai *et al*. (2024) revealed a significant main effect of vividness ($\beta$ = −0.32, SE = 0.065, *P* < .001) and VVIQ ($\beta$ = 0.13, SE = 0.066, *P* = .045). However, the interaction between VVIQ and vividness was not significant in this dataset ($\beta$ = −0.04, SE = 0.048, *P* = .36).

## Discussion

Perception and mental imagery rely on similar representations (Siclari *et al*. 2017; Dijkstra *et al*. 2019a; Pearson 2019). However, the extent to which they rely on similar appraisal mechanisms remains underexplored. A robust asymmetry in perceptual detection is that it takes longer to report that a stimulus is absent than to report that it is present. Here, we report a novel observation with respect to the appraisal of mental images: similar to perception, less vivid imagery takes longer to report than highly vivid imagery (Fig. 1). Furthermore, in certain datasets we show that this effect is strengthened both for individuals showing a greater asymmetry in response times in perceptual detection decisions, as well as those with greater trait imagery vividness (Fig. 2).

The relationship between reaction times in imagery and perception has been used previously to argue for a functional overlap between the two domains. For instance, it is known that during perception, foveated targets are subject to faster responses than those in the periphery (Chelazzi *et al*. 1988). The same has been shown for imagery, where participants are faster to report the formation of central mental images compared to those in the periphery (Marzi *et al*. 2006). Such findings lend support to models describing imagery as recruiting similar computational mechanisms to visual perception, specifically its retinotopic organization (Klein *et al*. 2004; Slotnick *et al*. 2005). Additionally, across a range of low-level visual features, response times for imagery and perception are correlated (Broggin *et al*. 2012). For instance, participants are faster to report the formation of mental images of shapes when they are of high, rather than low, luminosity, while the same pattern was observed during perceptual detection of such shapes. Here, we extend this line of work to incorporate the speed at which people report their imagery to be vivid or not and relate this to established behavioural signatures found in perceptual detection tasks.

The underlying mechanism that causes the symmetry in reaction time effects between imagery and perception remains unclear. Intuitively, one might believe that, since mental images are initiated internally and thus the mechanisms that determine an image’s qualities are also internal, we should have immediate access to its qualities from a direct read-out of the image-forming mechanisms. If this were the case, any differences in reaction time would be due to differences in other processes unrelated to the evaluation of mental images, such as the time it takes to generate the image. However, in our study, this would suggest a rather counterintuitive effect: it takes more time to form a weak image than to form a vivid image.

Alternatively, an explanation in terms of differences in evaluation time for weak and vivid images can be found in a recent theoretical account for the asymmetry in reaction time between target-present and target-absent responses (Mazor 2025; Mazor *et al*. 2025). According to this proposal, the reaction time is a product of distinct mechanisms for the evaluation of absence compared to presence. In the model, evidence for absence can never be obtained directly by perceptual systems but instead must be inferred from a lack of positive evidence for a target. This inference follows an implicit counterfactual reasoning process of the sort ‘if it had been present, I would have seen it’ (Mazor *et al*. 2025). One possibility is that similar inferential mechanisms are necessary for the appraisal of mental images. Our finding that weaker images take longer to report—and that this is related to detection asymmetries in one dataset—is in line with this view, as it implies that reporting weak (or absent) mental images incurs greater processing cost than vivid (or present) images, perhaps indicative of a reliance on a similar type of inference to detect an absence of imagery.

Interestingly, previous work has found a negative correlation between participants’ average imagery vividness and reaction times on a vividness task (Liu and Bartolomeo 2023), corroborating the main effect of VVIQ on reaction time we found in Cabbai *et al*.’s (2024) data. Whilst this between-subject effect is relevant to our understanding of how the overall vividness of one’s imagery impacts behavioural measures, it does not necessarily provide insight into the mechanisms responsible for the subjective appraisal of imagery and perception discussed here. Our conceptual model is focused on the within-subject evaluation of the vividness of imagery, which is made relative to participants’ internal criterion of what counts as vivid. As such, our results show that participants are faster to report when a mental image is vivid to them. Similarly, the reaction time asymmetries found in perception are all within-participants (Meuwese *et al*. 2014; Mazor *et al*. 2020, 2021, 2025; Kellij *et al*. 2021). In contrast, between-subject covariance of average speed of vividness ratings and trait imagery are likely to reflect different processes. Whilst vivid imagers may have greater evidence in favour of a vivid image on any given trial, they might also have a higher criterion of what counts as vivid given that they are used to vivid imagery. This may explain patterns in datasets where trial by trial vividness is related to both reaction times and VVIQ, but VVIQ and reaction times are not related themselves (Liu *et al*. 2025). As such, it does not necessarily follow that, since participants are faster to report vivid imagery, more vivid imagers should be faster overall. Alternatively, the VVIQ may be a less reliable self-report measure than trial-by-trial vividness ratings (Runge *et al*. 2017). Future work examining the relative contribution of vividness reports and VVIQ ratings to perceptual and imagery performance will be necessary to ascertain how different methods of report relate to perceptual and imagery experiences (e.g. Liu *et al*. 2025).

Similar reaction time profiles across perception and imagery may also be affected by non-perceptual biases. For example, vividness ratings are often presented on linear scales meaning that responses for weak imagery are presented on the left of the screen and vivid imagery on the right. This means that differences in motor speed for different response options could contaminate reaction times. We note, however, that in three of the datasets analyzed here (Dijkstra *et al*. 2019; Cabbai *et al*. 2023; Perception and Imagery Pilot Data) participants used a mouse to report their imagery vividness, meaning that disparities in reaction times for key presses from different fingers are unlikely to fully explain the effect of imagery vividness on reaction time. It is also possible that a participant’s arousal could drive the negative correlation between vividness and reaction time. For instance, if a participant has low arousal on a certain trial, they are perhaps also more likely to have both weaker imagery and a slower reaction time on that trial too. We note that detection asymmetries in perception persist even when trials are grouped according to the stimulus (present or absent) rather than the participants’ responses (Dijkstra et al. 2025; Pilot Data 2025; Mazor *et al*. 2025). This effectively orthogonalizes presence-absence comparisons from participants’ arousal. Developing objective measures of mental imagery will allow future experiments to decouple arousal levels and imagery vividness and fully determine the role of arousal in the reported effects.

The effect of perceptual and imagery vividness on reaction time may also be affected by a linguistic processing cost associated with negation (Wason 1959, 1961). The cost of negation is defined by longer reaction times when reading or evaluating sentences including negations (e.g. ‘not’, ‘without’, etc.), and this has even been shown to occur for sentences including words with negative semantics such as ‘fewer’, ‘hardly any’, or ‘a minority’ (Clark 1969; Just and Carpenter 1971). It is therefore possible that reporting an absence of perceptual visibility or ‘weak’ imagery may incur the same cost and may, to some extent, drive a negative correlation between reaction time and vividness reports in perception and imagery. It is worth noting, however, that detection asymmetries in reaction time emerge also when reports of absence do not correspond to verbal negation, such as when deciding whether a stimulus was a Q or an O (Mazor *et al*. 2021). More empirical work is needed to elucidate the underlying mechanism in the case of vividness judgements.

It is an open question to what extent this effect is invariant to the experimental designs chosen by researchers. Although the inverse correlation between reaction time and vividness was robust across all five datasets, the interaction of vividness and detection asymmetry only appeared in one of the two datasets that collected detection judgements. It is possible this is related to the difference in stimulus display across the two studies, although we note that the pilot dataset in which no interaction was found used online data with relatively few participants, which may have contributed both to this null finding and the disparity in reaction times seen across these two experiments.

Experimenter choices about how to prompt participants for vividness ratings may also impact results that rely on these subjective reports. The construct of vividness is conceptually difficult to define and operationalize in a precise way (Kind 2017), and participants are often left to interpret the notion themselves. Recent work has shown that ‘where’ imagery is experienced is not uniform across individuals, with relatively equal proportions of participants reporting imagery as either inside or outside the head (Schwarzkopf *et al*. 2025). This raises the possibility that different kinds of visualizers may call upon different features of imagery to determine vividness. For instance, imagers who experience images as projected into the external world may rely upon the precision of the image, whilst internal imagers may rely more on the accessibility of the image. Moreover, in certain clinical disorders, vividness judgements may not emerge from perceptual features at all and may instead rely on metacognitive processes (Kusztor *et al*. 2025). Our conceptual model may then predict varying strengths of correlation between reaction time and vividness across these different groups, with those who project their imagery perhaps showing the strongest relationship, owing to a greater similarity between imagery and external perception and thus relying more on perception-like evidence accumulation processes to determine the vividness of their imagery. Testing such a prediction could help delineate the computational and cognitive processes that underlie the potentially numerous ways people subjectively appraise their imagery, and would help make explicit the different dimensions of experience participants rely on when reporting their vividness (Fazekas *et al*. 2020; Fazekas 2024).

To conclude, across five independent datasets, we consistently found a negative correlation between imagery vividness judgements and the time taken to report imagery vividness, a behavioural signature corresponding to response time asymmetries in perceptual detection judgements. We suggest that this effect could be due to imagery employing a similar mechanism as perception to evaluate the strength of mental images. Experiments designed to reveal the true basis of this effect are warranted, and we hope that by drawing attention to this observation future models of perception and imagery can account for the robust relationship between vividness and reaction time.

## Data Availability

All data and scripts are available at https://osf.io/g7tjh/. The four published datasets can also be found in their original repositories: Dijkstra *et al*. 2019: https://data.donders.ru.nl, Cabbai *et al*. 2023: https://osf.io/wvnm8/, Cabbai *et al.* 2024: https://osf.io/y7a8c/, Dijkstra *et al*. 2025: https://github.com/ImagineRealityLab/NIMADET.
